# N-Acetyl Cysteine as a Novel Polymethyl Methacrylate Resin Component: Protection against Cell Apoptosis and Genotoxicity

**DOI:** 10.1155/2019/1301736

**Published:** 2019-09-15

**Authors:** Yu Zhang, Jian-feng Xiao, He-feng Yang, Yang Jiao, Wei-wei Cao, Huan-min Shi, Jing-fen Cun, Franklin R. Tay, Jie Ping, Yu-hong Xiao

**Affiliations:** ^1^The Affiliated Stomatological Hospital of Kunming Medical University, Kunming, China; ^2^Department of Stomatology, The 7th Medical Center of PLA General Hospital, Beijing, China; ^3^Freetech Technology, Nanjing, China; ^4^Department of Endodontics, The Dental College of Georgia, Augusta University, Augusta, GA, USA; ^5^Department of Medical Administration, The 7th Medical Center of PLA General Hospital, Beijing, China; ^6^Department of Stomatology, 920th Hospital of Joint Logistics Support Force, Kunming, China

## Abstract

The present study investigated the antiapoptotic and antigenotoxic capabilities of N-acetyl cysteine- (NAC-) containing polymethyl methacrylate (PMMA) resin. An *in vitro* Transwell insert model was used to mimic the clinical provisional restorations placed on vital teeth. Various parameters associated with cell apoptosis and genotoxicity were investigated to obtain a deeper insight into the underlying mechanisms. The exposure of human dental pulp cell (hDPC) cultures to the PMMA resin (Unifast Trad™) resulted in a rapid increase in reactive oxygen species (ROS) level beginning at 1 h, which was followed by time-dependent cell detachment and overt death. The formation of *γ*-H_2_AX and cell cycle G1 phase arrest indicated that oxidative DNA damage occurred as a result of the interactions between DNA bases and ROS, beyond the capacities of cellular redox regulation. Such oxidative DNA damage triggers the activation of p53 via the ataxia telangiectasia mutated (ATM) signaling pathway and the induction of intrinsic mitochondrial apoptosis. Oxidative stress, cell apoptosis, and DNA damage induced by the PMMA resin were recovered to almost the level of untreated controls by the incorporation of NAC. The results indicate that the PMMA resin induced the intrinsic mitochondrial apoptosis as a consequence of p53 activation via the ATM pathway in response to oxidative DNA damage. More importantly, the incorporation of NAC as a novel component into the Unifast Trad™ PMMA resin offers protective effects against cell apoptosis and genotoxicity. This procedure represents a beneficial strategy for developing more biocompatible PMMA-based resin materials.

## 1. Introduction

Provisional crowns and bridges are fabricated to protect tooth preparations and the adjacent gingiva until the insertion of permanent crowns. They are essential treatment procedures in fixed prosthodontics [1]. An acrylic-based self-polymerizing resin, primarily consisting of a prepolymerized polymethyl methacrylate (PMMA) powder and a liquid methyl methacrylate (MMA) monomer, is one of the most frequently used dental materials for this purpose [2]. Because the monomer-to-polymer conversion of the material is incomplete, unpolymerized monomers are released into the oral cavity, with the potential of interfering with the adjacent living oral tissues [3]. Both *in vitro* elution studies and clinical analyses of patient saliva after dental restorative procedures confirmed the release of resinous monomers after polymerization [4–8]. The chemical and biological effects of these resinous materials on dental pulp cells/tissues have been implicated after the diffusion of unpolymerized monomers through dentin, particularly in a clinical situation where the PMMA resin is loaded directly onto the prepared teeth [9, 10]. Previous studies have reported the toxicity and adverse effects of PMMA-based materials at both the tissue and cellular levels [11]. Although systemic toxicity has been rarely reported to date, local adverse effects including pulpal responses and oral hypersensitivity reactions, as well as fibrosis, necrosis, and histiocytosis in tissues around the materials have been clinically observed [12–14]. Investigations utilizing permanent cell lines or primary cultured cells derived from the dental pulp, gingiva, and periodontal ligament demonstrated that both the PMMA resin and the MMA monomer are cytotoxic via apoptosis, induce genotoxic effects, delay the cell cycle, and inhibit the mineralization processes [15–17]. Current findings strongly suggest that the mechanism behind these adverse effects involves the generation of oxidative stress [15]. It has been demonstrated that the exposure of cells to MMA, like other resin monomers, reduces the levels of the natural radical scavenger glutathione (GSH), while increasing the formation of reactive oxygen species (ROS) simultaneously [15]. High levels of ROS beyond the capacity of the body's inherent antioxidative mechanisms can react with cellular macromolecules, such as lipids, proteins, and DNA, via nonenzymatic glycation that results in progressive accumulation of advanced glycation end products in the tissues of the oral cavity [18, 19].

Based on the findings that oxidative stress is responsible for the toxic effects of resin materials, chemicals for scavenging ROS and antioxidants such as N-acetyl cysteine (NAC) have been identified to protect against related cell damage [20–23]. The effectiveness of NAC in preventing MMA-induced oxidative stress and apoptotic cell death has been also demonstrated in human dental pulp cells (hDPCs) [20]. As a cell-permeable cysteine derivative and an important precursor of GSH, NAC not only acts as a direct ROS scavenger but also promotes the intracellular GSH redox cycle [24]. The authors recently demonstrated that the addition of NAC at a specific mass fraction (0.15 or 0.30%) reduces cytotoxicity, with no significant deterioration in the mechanical properties of the PMMA resin [25]. Although some previous studies evidenced that a method of incorporating NAC into the PMMA resin materials improved biocompatibility, the underlying mechanism is still unknown [26, 27]. In addition, the potential of NAC in counteracting the adverse apoptotic and genotoxic effects of PMMA is of great clinical relevance but has not been thoroughly evaluated.

**Figure 1:**
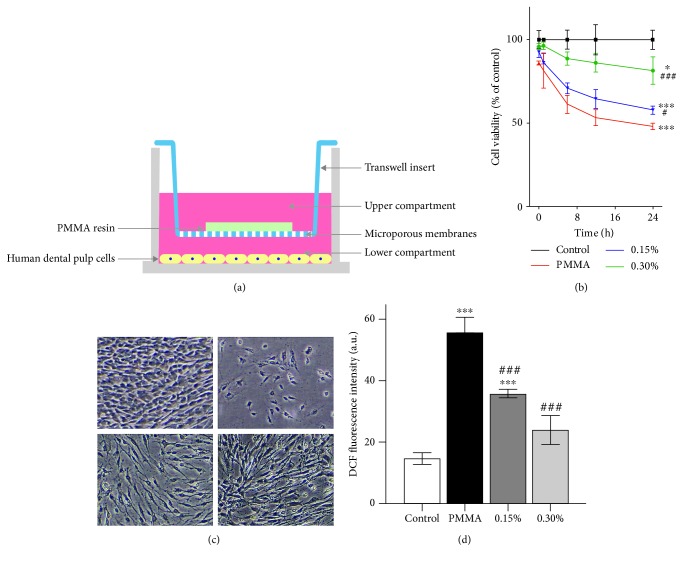
PMMA-induced ROS accumulation triggers cell death and its prevention by NAC incorporation. (a) Schematic representation of the coculture of hDPCs with the experimental PMMA resin specimens in the Transwell system. Experimental PMMA resin specimens were placed in the inserts (upper compartments), and hDPCs were seeded in the wells (lower compartments). (b) Viability of hDPCs as examined by the CCK-8 assay. (c) Visualization of hDPC viability observed by phase contrast microscopy after a 24 h exposure period. (d) Intracellular ROS level evaluated by flow cytometry after a 1 h exposure period. Data represent mean ± standard deviations (*n* = 3). ^∗^*P* < 0.05, ^∗∗^*P* < 0.01, and ^∗∗∗^*P* < 0.001 vs. untreated cells (control group); ^#^*P* < 0.05, ^##^*P* < 0.01, and ^###^*P* < 0.001 vs. PMMA-treated cells. Data were analyzed using one-way analysis of variance (ANOVA) or two-way ANOVA and post hoc Tukey's test.

Accordingly, the present study is aimed at providing more experimental evidence for the development of a more biocompatible PMMA resin by incorporating NAC as a component. The null hypothesis tested was that the incorporation of NAC exerts no detoxifying effects on PMMA-induced cell apoptosis and genotoxicity. An *in vitro* Transwell insert model, which allows the sharing of conditioned media without cell-substrate (hDPCs-experimental PMMA resins) contact, was used to mimic the clinical situation of the application of provisional restorations on vital teeth (Figure 1(a)). Primary hDPCs were examined for their cell viability and morphology, intracellular ROS level, cell apoptosis, caspase-3 activity, mitochondrial membrane potential (MMP), DNA damage, *γ*-H_2_AX immunofluorescence, and cell cycle upon PMMA resin exposure. The mRNA expression of genes related to cell apoptosis and DNA damage was also analyzed for mechanistic experiments.

## 2. Materials and Methods

### 2.1. Resin Preparation

The experimental PMMA resins were prepared as previously described, with minor modifications [25]. Briefly, untreated control PMMA resin (Unifast Trad™, GC Corp., Tokyo, Japan) was prepared by mixing the powder and liquid components for 30 s according to the manufacturer's instructions (powder/liquid ratio: 1.0 g/0.5 mL). N-acetyl cysteine (Sigma-Aldrich, St Louis, MO, USA) was prepared as 1.0 mol/L stock solution in double-distilled water, with pH adjusted to 7.2. The NAC-incorporated PMMA resin was prepared by mixing the powder and liquid containing the NAC solution. The concentration of NAC was set at 0.15 or 0.30% of the final resin substrate. The materials were placed in stainless steel molds and covered with glass slides. After setting for 30 min at 25°C, the specimens (6 mm diameter and 2 mm thick; *n* = 3) were rinsed with double-distilled water once.

### 2.2. Cell Culture and Treatment

The hDPCs were obtained from young healthy human subjects (18–25 years old) who had their noncarious third molars extracted. The procedure was reviewed and approved by the Ethics Committee of the 7th Medical Center of PLA General Hospital (approval no. 2019-037). After removal of dental pulp tissues from the tooth, the hDPCs were isolated and expanded as described in previous studies [20, 23, 25, 28]. Briefly, the dental pulp tissues were minced and digested in a solution containing 3 mg/mL type I collagenase and 4 mg/mL dispase (Gibco, Grand Island, NY, USA) at 37°C for 2 h. A single-cell suspension was obtained by passing the cells through a 70 mm strainer (BD Falcon, Franklin Lakes, NJ, USA). The harvested cells were cultured in *α*-minimal essential medium (*α*-MEM; Gibco) supplemented with 10% fetal bovine serum (FBS; Gibco), 2 mM L-glutamine, 100 U/mL penicillin, and 100 g/mL streptomycin (Invitrogen, Carlsbad, CA, USA) at 37°C in a humidified atmosphere of 5% CO_2_. The culture medium was changed every 3 days, and passage 2–4 cells were used for subsequent experiments.

For the studies described in the succeeding subsections, the hDPCs were cocultured with the specimens in Transwell systems (BD Falcon). The hDPCs were seeded in 24-well plates at a density of 2 × 10^4^ cells per well at 37°C in a humidified atmosphere of 5% CO_2_. When the cell growth achieved 70–80% confluence, the specimens were placed separately in Transwell inserts (3 mm pore size) with the hDPCs preincubated in the lower chambers. The system was incubated for the indicated times. The incubation times in the present work were selected based on pilot experiments. The ratio between the surface of the samples and the volume of the medium was 1.25 cm^2^/mL according to ISO standards (10993-12:2007).

### 2.3. Cell Viability and Morphology

Cell viability was determined using the Cell Counting Kit-8 (CCK-8; Beyotime, Jiangsu, China) according to the manufacturer's instructions. Briefly, hDPCs were seeded into 96-well plates at 5 × 10^3^ cells per well with a group of blank control wells (without cells) and a group of untreated control wells (cells exposed only to culture medium). When the cells achieved 70–80% confluence, 200 *μ*L of the cell culture from each of the Transwell systems were transferred to 96-well plates and incubated for the indicated time periods. Each incubation was performed in three separate cell culture wells. CCK-8 solution of 20 *μ*L was then added, and the cells were incubated at 37°C for another 4 h. Cell viability was obtained by monitoring the color change with a microplate reader (Bio-Rad, Hercules, CA, USA) with absorbance read at 450 nm. Percentage cell viability was determined by the following formula: optical density (OD; treated sample − blank)/OD (untreated sample − blank) × 100%. Phase contrast images were acquired using an IX70 microscope (Olympus, Tokyo, Japan). Three independent fields were acquired for each experimental condition. Representative samples from one field of view were shown.

### 2.4. Formation of Reactive Oxygen Species (ROS)

Production of ROS was determined using an ROS Assay Kit (Beyotime) according to the manufacturer's instructions. Briefly, hDPCs were incubated with 2′7′-dichlorodihydrofluorescin diacetate prior to harvesting with PBS/EDTA. Analysis was performed with flow cytometry (FACScan, Becton Dickenson, San Jose, CA, USA). A minimum of 10^4^ cells were analyzed for each sample. Fluorescence intensity values were normalized to the fluorescence detected in the untreated control culture samples.

### 2.5. Caspase-3 Activity

Caspase-3 activity was determined using a Colorimetric Caspase-3 Assay Kit (Beyotime) according to the manufacturer's instructions. Briefly, hDPCs were lysed using a lysis buffer (Beyotime). Protein concentration was measured using a BCA Protein Assay Kit (Beyotime). Cell lysates, reaction buffer, and substrate (Ac-DEVD-pNA) were added to each tube. After incubation at 37°C for 2 h, the samples were measured with a microplate reader (Bio-Rad) with absorbance read at 450 nm. Caspase enzymatic activities in the cell lysates were directly proportional to the color reaction and were normalized to protein contents.

### 2.6. Mitochondrial Membrane Potential

Mitochondrial membrane potential (MMP) was determined using the fluorescent dye JC-1 (Beyotime) according to the manufacturer's instructions. Briefly, harvested hDPCs were incubated with the JC-1 staining solution and then rinsed with JC-1 staining buffer. Emission filters of 535 and 595 nm were used to quantify the population of mitochondria with green (JC-1 monomers) and red (JC-1 aggregates) fluorescence by flow cytometry (FACScan). A minimum of 10^4^ cells were analyzed per condition.

### 2.7. Detection of Apoptosis and Necrosis

Apoptosis and necrosis were determined using an Annexin V-FITC Apoptosis Detection Kit (Beyotime) according to the manufacturer's instructions. Briefly, hDPCs were harvested with PBS/EDTA and collected by centrifugation. Cells in apoptosis or necrosis were identified after staining with annexin V-FITC and propidium iodide (PI), and fluorescence was analyzed by flow cytometry (FACScan). A minimum of 10^4^ cells were analyzed for each sample. To further investigate the function of Nrf2, hDPCs were pretreated with or without 25 *μ*M *tert*-butylhydroquinone (tBHQ), a Nrf2 activator, for 18 h and subsequently cotreated with the PMMA resin.

### 2.8. DNA Damage Analysis

DNA damage was determined using the EpiQuik™ *In-Situ* DNA Damage Assay Kit (EpiGentek, Farmingdale, NY, USA) according to the manufacturer's instructions. Absorbance was read on a microplate reader (Bio-Rad) at 450 nm. Percentage DNA damage was determined by the following formula: OD (treated sample − blank)/OD (untreated sample − blank) × 100%.

### 2.9. *γ*-H_2_AX Immunofluorescence

The *γ*-H_2_AX immunofluorescence assay was performed using the OxiSelect DNA Double-Strand Break Staining Kit (Cell Biolabs Inc., San Diego, CA, USA) according to the manufacturer's instructions. Briefly, hDPCs were fixed with paraformaldehyde (3.7%) and incubated with blocking buffer on an orbital shaker. The cells were then incubated with an anti-phospho-histone (*γ*-H_2_AX) antibody solution, followed by a secondary antibody and a Cy3 conjugate solution. The nuclei were stained with Hoechst 33342 (Sigma-Aldrich). Fluorescent images were obtained using laser scanning confocal microscopy (Keyence Corp., Osaka, Japan).

### 2.10. Cell Cycle Analysis

Cell cycle was determined using a Cell Cycle and Apoptosis Analysis Kit (Beyotime). Briefly, harvested hDPCs were treated with a mixed solution of RNase and PI, and fluorescence was analyzed with flow cytometry (FACScan). A minimum of 10^4^ cells were analyzed for each sample.

### 2.11. Gene Expression Analysis

Total RNA was isolated using the TRIzol Reagent (Invitrogen) and reverse transcribed to complementary DNA (cDNA) using the PrimeScript RT Reagent Kit (TaKaRa, Dalian, China). Quantitative real-time quantitative polymerase chain reaction (qRT-PCR) was performed using SYBR Premix Ex Taq II (TaKaRa) in the Bio-Rad CFX96™ Real-Time PCR Detection System. The resulting amplification and melt curves were analyzed to ensure the identity of the specific PCR product. Threshold cycle values were used to calculate the fold change in the transcript levels by using the ^ΔΔ^CT method. Primers are listed in Table 1. The housekeeping gene *GAPDH* was used to normalize the expression level of related genes.

### 2.12. Statistical Analysis

All values were presented as the mean ± standard deviation (SD) from three independent experiments. Comparisons among groups were analyzed with GraphPad Prism 5 software (San Diego, CA, USA) using one-way analysis of variance (ANOVA) or two-way ANOVA and post hoc Tukey's test. Significant differences were considered when *P* < 0.05.

## 3. Results

### 3.1. PMMA Triggers Oxidative Cell Death and Its Prevention by NAC

The exposure of hDPCs to the PMMA resin using a Transwell insert model resulted in a time-dependent reduction in cell viability (Figure 1(b)) as well as cell detachment (Figure 1(c)). For example, cell viability was reduced to 48% by the PMMA resin compared to untreated cell cultures after a 24 h incubation. Conversely, NAC incorporation significantly protected hDPCs against PMMA-induced cell death in a concentration-dependent manner. Cell viability increased to 57.7% and 81.4% in cell cultures exposed to the PMMA resin supplemented with 0.15 and 0.30% NAC, respectively. Concomitantly, ROS level was significantly elevated in cell cultures exposed to the PMMA resin, as detected by DCF fluorescence after a 1 h exposure period (Figure 1(d)). By contrast, ROS accumulation was suppressed by NAC incorporation at both tested concentrations.

### 3.2. Reversal of PMMA-Induced Cell Apoptosis by NAC

**Figure 2:**
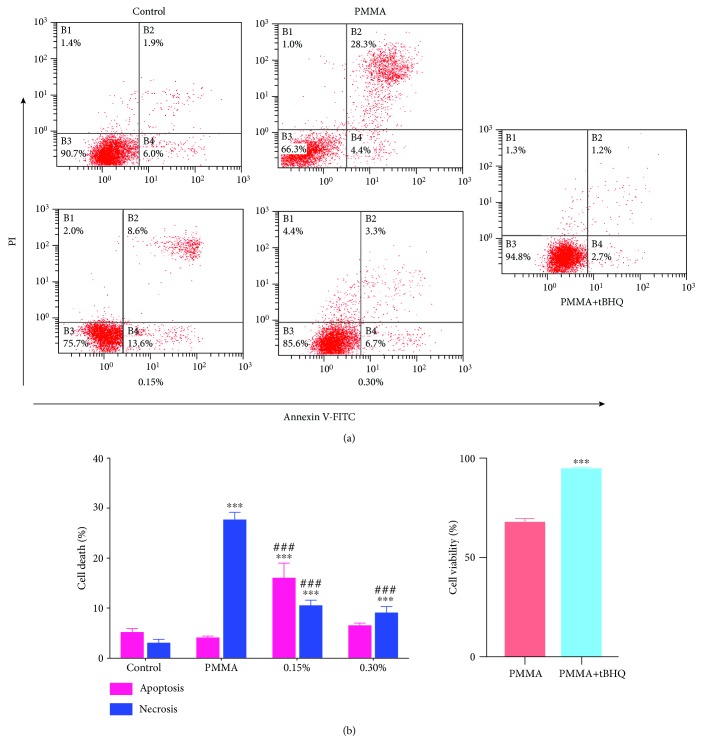
Induction of apoptosis and necrosis in PMMA-treated hDPCs. After a 24 h exposure period, cells were stained with annexin V-FITC/propidium iodide (PI) and analyzed by flow cytometry. (a) Percentages of viable cells (unstained, B3) and cells in apoptosis (annexin, B4), late apoptosis (annexin and PI, B2), and necrosis (PI, B1) of one typical experiment are denoted in the quadrants of each density blot. (b) Bar graphs represent flow cytometry data. Data represent mean ± standard deviations (*n* = 3). ^∗^*P* < 0.05, ^∗∗^*P* < 0.01, and ^∗∗∗^*P* < 0.001 vs. untreated cells (control group); ^#^*P* < 0.05, ^##^*P* < 0.01, and ^###^*P* < 0.001 vs. PMMA-treated cells. Data were analyzed using one-way analysis of variance (ANOVA) and post hoc Tukey's test.

**Figure 3:**
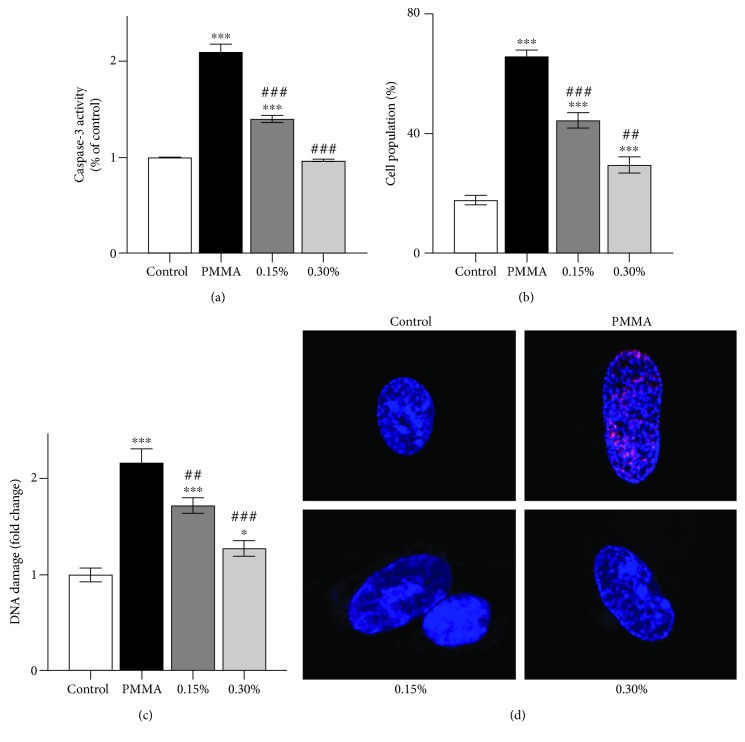
Caspase-3 activity, mitochondrial membrane potential, and genotoxicity in PMMA-treated hDPCs. (a) Caspase-3 activity, (b) mitochondrial membrane potential, and (c) DNA damage in hDPCs exposed to untreated control or NAC-incorporated PMMA resins after a 24 h exposure period. Data represent mean ± standard deviations (*n* = 3). ^∗^*P* < 0.05, ^∗∗^*P* < 0.01, and ^∗∗∗^*P* < 0.001 vs. untreated cells (control group); ^#^*P* < 0.05, ^##^*P* < 0.01, and ^###^*P* < 0.001 vs. PMMA-treated cells. Data were analyzed using one-way analysis of variance (ANOVA) and post hoc Tukey's test. (d) Representative images of immunofluorescent staining for *γ*-H2AX in hDPCs exposed to untreated control or NAC-incorporated PMMA resins for 24 h. Hoechst 33342 (blue fluorescence) is a marker for DNA and stains the whole nucleus of a cell, while the red fluorescence indicates *γ*-H2AX-specific foci. (a) A nucleus of hDPCs without foci is typically seen in untreated cells. (b) A nucleus of hDPCs with typical *γ*-H2AX-specific foci can be observed in PMMA-treated cells. (c and d) NAC incorporation at both mass fractions reduced the red *γ*-H2AX-specific foci in the nucleus of hDPCs exposed to PMMA.

The nature of PMMA-induced cell death was investigated by staining hDPCs with annexin V/PI, and fluorescently labelled cells were analyzed by flow cytometry. As shown in Figures 2(a) and 2(b), untreated cell cultures manifested minimal signs of apoptotic or necrotic cell death, with more than 91% of the cells remaining viable. Conversely, exposure of cell cultures to the PMMA resin significantly reduced the percentage of viable cells to 68.1% and increased the number of cells in the various phases of cell death, especially late apoptosis (27.1%). The concentration-dependent increase in the percentage of viable cells was indicative of a well-defined protective effect of NAC on PMMA-induced programmed cell death (73.3% by 0.15% and 84.3% by 0.30% NAC). The incorporation of NAC shifted the mode of cell death caused by the PMMA resin from necrosis to apoptosis. Although the number of cells in necrosis (PI) and late apoptosis (annexin and PI) in PMMA-exposed cell cultures decreased after the incorporation of NAC, the percentage of apoptotic cells (annexin) increased in parallel. To further define the role of oxidative stress in PMMA-induced cell death, we investigated the involvement of redox-sensitive transcription factor nuclear factor erythroid 2-related factor 2 (Nrf2). PMMA-induced apoptosis was completely inhibited by the pretreatment of the Nrf2 activator tBHQ (cell viability of 95.1% *vs.* 68.1%; Figure 2(c)). The occurrence of apoptosis was further confirmed by the increase in caspase-3 activity (Figure 3(a)) and MMP depolarization (Figure 3(b)) in cell cultures exposed to the PMMA resin. These effects were considerably reduced by NAC incorporation into the PMMA resin.

### 3.3. Protection against PMMA-Induced Genotoxicity and Cell Cycle Arrest by NAC

Excessive ROS interacts with cellular biomolecules such as DNA and may cause damage to DNA bases. Thus, the phosphorylation of H_2_AX to *γ*-H_2_AX, which is one of the earliest chromatin modification events in DNA damage response, was detected by a commercial immune-enzymatic colorimetric assay. As shown in Figure 3(c), significant DNA damage was detected in cell cultures exposed to the PMMA resin, compared to untreated control cultures after a 24 h exposure period. Such an effect was reduced by NAC regardless of incorporated mass fraction. Similar results were revealed by immunofluorescence staining (Figure 3(d)). The red fluorescence of *γ*-H_2_AX was readily discernible in hDPC nuclei by immunofluorescence using *γ*-H_2_AX-specific antibodies. Microscopic examination showed that PMMA resin exposure induced *γ*-H2AX-specific foci, while the incorporation of NAC weakened such an effect.

**Figure 4:**
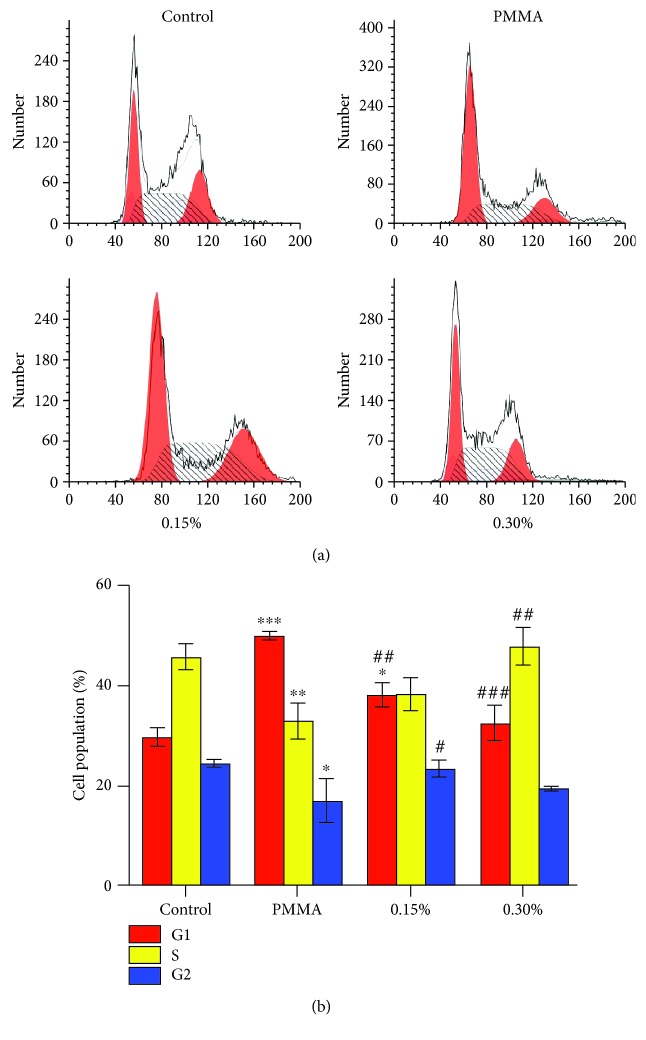
PMMA-induced cell cycle arrest in hDPCs. (a) Cell cycle analysis of hDPCs exposed to untreated control or NAC-incorporated PMMA resins after a 24 h exposure period. (b) Bar graphs represent flow cytometry data. Data represent mean ± standard deviations (*n* = 3). ^∗^*P* < 0.05, ^∗∗^*P* < 0.01, and ^∗∗∗^*P* < 0.001 vs. untreated cells (control group); ^#^*P* < 0.05, ^##^*P* < 0.01, and ^###^*P* < 0.001 vs. PMMA-treated cells. Data were analyzed using one-way analysis of variance (ANOVA) and post hoc Tukey's test.

Damage of DNA may result in the activation of cell cycle checkpoints [29]. Accordingly, the effect of the experimental PMMA resin on the cell cycle of hDPCs was analyzed after an exposure period of 24 h (Figures 4(a) and 4(b)). The number of cells in the G1 phase increased from 30% to 50%, while the cell numbers in the S phase decreased from 45% in untreated cultures to 33% in cell cultures treated with the PMMA resin. A dose-dependent recovery of the PMMA resin-induced cell cycle arrest was observed in cell cultures exposed to the PMMA resin supplemented with both 0.15 and 0.30% NAC.

### 3.4. Recovered Expression of Apoptosis- and DNA Damage-Related Genes by NAC

**Figure 5:**
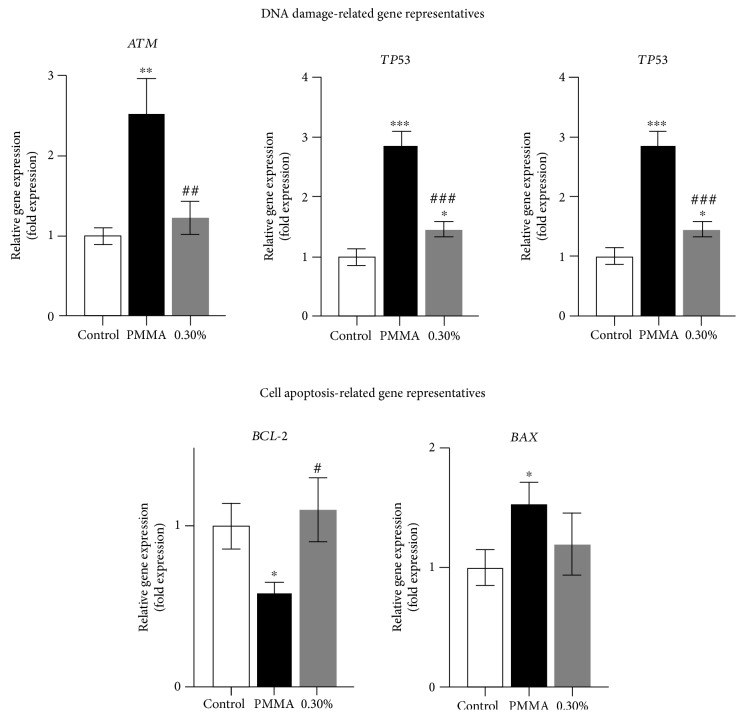
PMMA-induced DNA damage- and cell apoptosis-related gene expression in hDPCs. Gene expression of *ATM*, *BCL-2*, *BAX*, *P21*, and *TP53* in hDPCs exposed to untreated control or NAC-incorporated PMMA resins after a 24 h exposure period. Data represent mean ± standard deviations (*n* = 3). ^∗^*P* < 0.05, ^∗∗^*P* < 0.01, and ^∗∗∗^*P* < 0.001 vs. untreated cells (control group); ^#^*P* < 0.05, ^##^*P* < 0.01, and ^###^*P* < 0.001 vs. PMMA-treated cells. Data were analyzed using one-way analysis of variance (ANOVA) and post hoc Tukey's test.

The mRNA expression of genes related to cell apoptosis and DNA damage was analyzed for mechanistic purposes (Figure 5). The upregulation of *ATM*, *BAX*, *P21*, and *TP53* was significantly induced in cell cultures treated with the PMMA resin, whereas the expression of *BCL-2* was downregulated compared to untreated control cultures after a 24 h exposure. The altered gene expressions by the PMMA resin was significantly recovered after the incorporation of NAC.

## 4. Discussion

The present study represents the first investigation that evaluated the effects of NAC, as a component of the PMMA resin, on the cell apoptosis and genotoxicity of the carrier material. For decades, NAC has been used for the treatment of many clinical diseases such as cystic fibrosis, acetaminophen poisoning, doxorubicin-induced cardiotoxicity, and heavy metal toxicity [24]. With respect to dentistry, NAC has been explored to prevent the cytotoxic effects of resin monomers [15]. Recently, the authors demonstrated that incorporation of 0.15 or 0.30% NAC reduces cytotoxicity without compromising the mechanical properties of the PMMA resin [25]. Based on these mass fractions, the present study was designed to provide more experimental evidence regarding the potential antiapoptotic and antigenotoxic activities of the novel PMMA resin.

Provisional restorations using the self-curing PMMA dental resin directly onto the prepared vital teeth create a clinical situation crucial for physiological responses in dental pulp cells. Depending on the remaining dentin thickness in deep cavities, small and hydrophilic monomer MMA and other compounds released from unpolymerized resinous materials may diffuse across a thin remaining dentin layer and trigger adaptive cell responses in pulp tissue. Thus, different from previous studies that cultured the tested cells directly on the PMMA resin or using resin extracts for *in vitro* cell experiments [25, 27, 30–32], a Transwell insert model was utilized in this study to mimic this clinical situation. The hDPCs from the primary culture were used as a suitable model cell [25]. The exposure of cell cultures to the PMMA resin resulted in a time-dependent cytotoxic effect in these cells, as repeatedly established in related recent projects [25, 26, 33]. Such toxicity may be caused by the leaching of the MMA monomer and additives due to incomplete polymerization. Resin monomers cause cytotoxicity via numerous mechanisms, which are causally associated with oxidative stress, ROS generation, and apoptosis [15]. The beneficial effect of NAC, as demonstrated in the present study, also confirmed a critical role of ROS in the cytotoxic response. However, high NAC concentrations were reported to reduce the critical physiological levels of ROS which are necessary to act as signals essential for normal cell vitality and proliferation [34]. The accumulation of intracellular ROS is the main characteristic of oxidative stress, and it is the first major cellular response toward environmental insults, including resin monomers. Reactive oxygen species were detected after a short exposure of the cell cultures to the PMMA resin. It has been well established that oxidative stress is capable of damaging major cellular functions because of lipid peroxidation, as well as protein and DNA oxidation. In the authors' previous study, differential changes in the activities of antioxidative enzymes such as superoxide dismutase (SOD), glutathione peroxidase (GPx), and catalase (CAT), as well as increased content of the lipid peroxidation product malondialdehyde were detected in cells exposed to the MMA monomer [20]. Noteworthy is that the expression of these antioxidative enzymes is under the control of Nrf2 [35], and it has recently been demonstrated that the activation of Nrf2-regulated cellular antioxidative defense inhibited resin monomer-induced oxidative stress and supported cell viability [36, 37]. Consistent with these studies, our findings showed that the pharmacological activation of Nrf2 by tBHQ protected the cells from PMMA-induced apoptosis, providing an alternative effective therapeutic target to maintain vital physiological functions under monomer-induced oxidative stress conditions.

The induction of oxidative DNA damage has been directly related to a variety of chronic degenerative diseases such as Alzheimer's disease, cancer, and rheumatoid arthritis [38]. Phosphorylation of histone H_2_AX to *γ*-H_2_AX at the sites of DNA damage is an early step in the cellular response to DNA double-strand breaks (DSBs). In the present study, *γ*-H_2_AX was detected using both an immune-enzymatic colorimetric assay and immunofluorescence staining. Moreover, extensive evidences indicate the formation of DNA DSBs in resin monomer-exposed cells [39, 40]. Cells respond to DNA damage by blocking the cell cycle and activating the cell cycle checkpoints to allow for DNA repair. Here, the leached MMA monomer caused a corresponding delay of the cell cycle in the G1 phase. This observation would also indicate that checkpoints in the G1 phase are very sensitive to DNA damage under the current experimental conditions [41]. On the other hand, the finding of the protective role of NAC incorporation provides further evidence that oxidative DNA damage is likely the origin of resin monomer-induced cell cycle delay.

Ataxia telangiectasia mutated (ATM) protein can be recruited and activated by DNA DSBs, which further activates p53 [29]. As the guardian of the genome, p53 is a key regulatory protein of the cell cycle, DNA repair, and the induction of apoptosis caused by DNA damage [42]. In response to oxidative stress, p21 which is tightly controlled by p53, is activated to function as an inhibitor of cell cycle progression at the G1 phase [43]. Thus, the authors hypothesized that the ATM-related signaling pathway might be involved in PMMA resin-induced cell damage. In the present study, expressions of *ATM*, *P21*, and *TP53* were significantly increased in cell cultures exposed to the PMMA resin. Furthermore, the causal relationship between the generation of oxidative stress and the activation of ATM became even more apparent, since the altered expression of the aforementioned genes was substantially recovered after the incorporation of NAC. Therefore, the genotoxicity of PMMA resin- and MMA monomer-induced apoptosis may be initiated by the cellular response to oxidative DNA damage followed by the activation of a communicating network of pathways involving ATM, p53, and p21 [44]. Similar results were observed recently in other dental resin monomers [28, 45]. The activation of p53 by ATM further transcriptionally stimulates the expression of pro- and antiapoptotic proteins of the Bcl-2 family and unleashes the enzymatic apoptotic machinery of caspases. The qRT-PCR analyses conducted in the present work showed the downregulation of *BCL-2* expression as well as the upregulation of *BAX* expression after the cell cultures were exposed to the PMMA resin for 24 h. The occurrence of apoptosis was further confirmed by the increase in caspase-3 activity and MMP depolarization. These obtained results correspond with our previous findings using western blot analysis which showed that the expression of Bcl-2 was downregulated, whereas the expression of Bax and cleaved caspase-3 were upregulated by the MMA monomer, which is a major component leaching from the PMMA resin and exerting adversely biological effects [20]. Thereafter, the Bcl-2 family of proteins change their usual localization as well as targeting patterns, trigger mitochondrial dysfunction, MMP depolarization, cytochrome C release, and caspase activation, and eventually cause cell death via apoptosis [20, 45]. Based on the findings of the present study, it is demonstrated that the PMMA resin induced the intrinsic mitochondrial apoptosis as a consequence of p53 activation via ATM signaling upon oxidative DNA damage.

**Figure 6:**
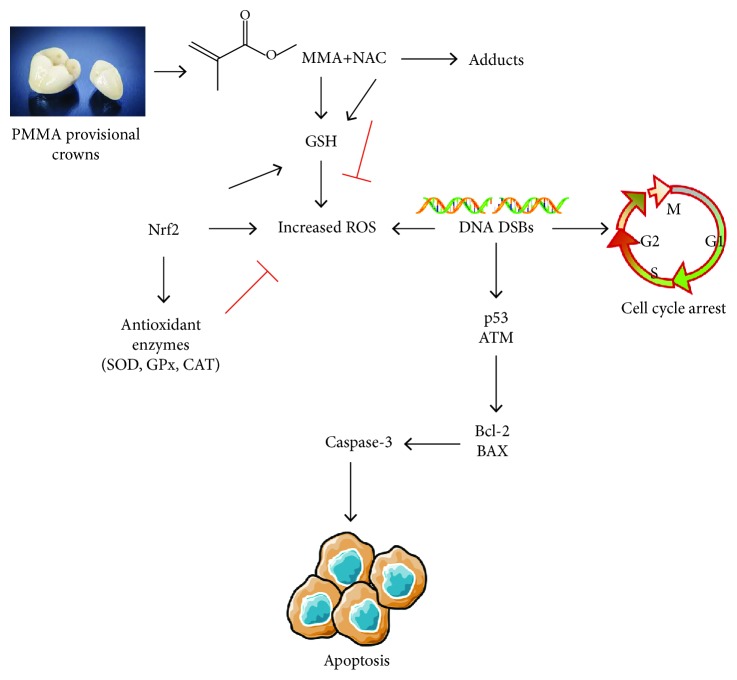
Schematic model. The PMMA resin and the MMA monomer lead to the formation of increased levels of ROS after the depletion of GSH, which in turn activates the Nrf2-mediated antioxidant system and differential expression of antioxidative enzymes like SOD, GPx, and CAT, to protect cells from oxidative stress. However, oxidative stress beyond the capacities of cellular redox regulation causes oxidative DNA damage and cell cycle arrest, which subsequently upregulates p53 via ATM signaling and triggers intrinsic mitochondrial apoptosis. NAC alleviates PMMA-induced oxidative stress and protects cells from apoptosis by intracellular ROS scavenging, GSH replenishment, and direct chemical reaction to reduce the availability of a free monomer. This illustration summarizes the Discussion and is based on the current data and previous studies.

In this study, we further found that the incorporation of NAC, especially at 0.30%, protected cells against PMMA resin-induced toxic effects. Such a procedure almost completely blocked the aforementioned molecular signaling pathways that result in resin monomer-induced cell death. To the best of our knowledge, this is the first report demonstrating that NAC incorporation protects against PMMA resin-induced hDPC apoptosis and genotoxicity. Consequently, the null hypothesis has to be rejected. Mechanically, it was believed that NAC exerts protective effects against resin monomer-induced cytotoxicity possibly by intracellular ROS scavenging and GSH replenishment [20, 45, 46]. Recent studies showed that NAC also reduces the availability of free dental resin monomers by direct chemical reaction with their methacrylic group [23, 47, 48] (Figure 6). Because of the strong ability of scavenging radicals to interfere with the diffusion of electrons, incorporation of NAC impedes the polymerization of the organic resin matrix. A dose-dependent decrease in the degree of conversion was identified for the investigated PMMA resin containing NAC, when compared to the control [25]. However, a recent study reported a significant reduction in the release of resin monomers after the incorporation of NAC at specific mass ratios [49]. This result may be explained by the interaction of NAC with resin components, and consequently, less monomers are available for leaching. Accordingly, the authors hypothesized that the incorporation of NAC as a resin component may achieve clinical benefits such as better biocompatibility and acceptable mechanical properties. Future investigations concerning its effects in a clinical setting will be of great interest.

## 5. Conclusion

Incorporation of NAC into the Unifast Trad™ PMMA resin achieves protective effects against cell apoptosis and genotoxicity. This procedure represents a beneficial strategy for developing resin materials with better biocompatibility.

## Figures and Tables

**Table 1:** Primer sequences.

Gene	Forward (5′–3′)	Reverse (5′–3′)
*ATM*	TGGTGCTATTTACGGAGCTG	TTCGAAAGTTGACAGCCAAA
*P21*	GTTCCTTGTGGAGCCGGAGC	GGTACAAGACAGTGACAGGTC
*TP53*	CGGAGGTCGTGAGACGCTG	CACATGTACTTGTAGTGGATGGTGG
*BCL-2*	CCTGTGGATGACTGAGTACCTGAAC	CAGAGTCTTCAGAGACAGCCAGGA
*BAX*	CAGGATGCGTCCACCAAGAA	GCAAAGTAGAAGAGGGCAACCAC
*GAPDH*	ATGACATCAAGAAGGTGGTG	CATACCAGGAATGAGCTTG

## Data Availability

The data used to support the findings of this study are available from the corresponding authors upon request.
