# Corrigendum to “N-Acetyl Cysteine as a Novel Polymethyl Methacrylate Resin Component: Protection against Cell Apoptosis and Genotoxicity”

**DOI:** 10.1155/2020/2768238

**Published:** 2020-09-26

**Authors:** Yu Zhang, Jian-feng Xiao, He-feng Yang, Wei-wei Cao, Huan-min Shi, Jing-fen Cun, Franklin R. Tay, Jie Ping, Yang Jiao, Yu-hong Xiao

**Affiliations:** ^1^The Affiliated Stomatological Hospital of Kunming Medical University, Kunming, China; ^2^Freetech Technology, Nanjing, China; ^3^Department of Endodontics, The Dental College of Georgia, Augusta University, Augusta, GA, USA; ^4^Department of Medical Administration, The 7th Medical Center of PLA General Hospital, Beijing, China; ^5^Department of Stomatology, The 7th Medical Center, Chinese PLA General Hospital, Beijing, China; ^6^Department of Stomatology, 920th Hospital of Joint Logistics Support Force, Kunming, China

In the article titled “N-Acetyl Cysteine as a Novel Polymethyl Methacrylate Resin Component: Protection against Cell Apoptosis and Genotoxicity” [1], there was an error in Figure 5. The qRT-PCR results of the TP53 gene were mistakenly duplicated, and the figure should have included the qRT-PCR results of the P21 gene. The authors apologize for this error, and the corrected Figure 5 is shown below:

## Figures and Tables

**Figure 1:**
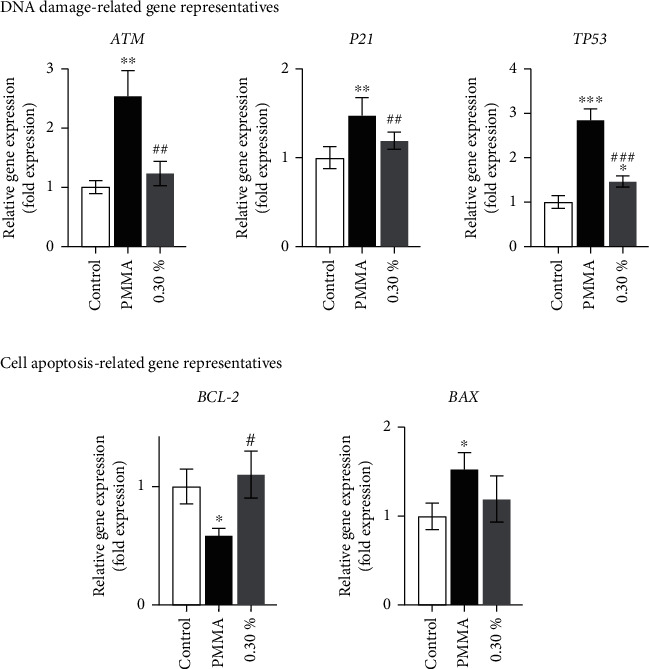
PMMA-induced DNA damage- and cell apoptosis-related gene expression in hDPCs. Gene expression of ATM, BCL-2, BAX, P21, and TP53 in hDPCs exposed to untreated control or NAC-incorporated PMMA resins after a 24 h exposure period. Data represent mean ± standard deviations (*n* = 3). ^∗^*P* < 0.05, ^∗∗^*P* < 0.01, and ^∗∗∗^*P* < 0.001 vs. untreated cells (control group); ^#^*P* < 0.05, ^##^*P* < 0.01, and ^###^*P* < 0.001 vs. PMMA-treated cells. Data were analyzed using one-way analysis of variance (ANOVA) and post hoc Tukey's test.
